# Hospital Statistics

**Published:** 1859-12

**Authors:** 


*Hospital Statistics.*—Guy's Hospital, founded by Thomas Guy, in 1721, for the reception of 400 patients, and recently enlarged through the aid of a large bequest from the late William Hunt, contains at the present time nearly 550 beds; and, with its extensive buildings and airing grounds, occupies an area of about seven acres. The hospital is divided into medical, surgical, clinical, ophthalmic, uterine, and venereal wards, independently of a ward, in a detached building, for lunatic patients, the vacancies in which the governors of the hospital have of late years forborne to fill up. In the year 1857, 44,281 persons were relieved by its means; 5,226 as in-patients, 9,889 as out-patients, and 25,886 as casualties, besides 1,731 women who were attended in their confinements, and 1,540 who received advice from the Lying-in Charity. Four hundred patients are now received into the original building of Guy, and one hundred and fifty into the part of the new wing already completed; the latter building, when finished, will admit three hundred persons.

St. Bartholomew's Hospital contains 650 beds, of which 420 are allotted to surgical cases and diseases of the eye, and 230 to medical cases and the diseases of women. The number of patients is more than 95,000 annually; the in-patients amounting to upward of 6,000, the out-patients and casualties to more than 89,000.

The London Hospital contains 445 beds, of which 135 are allotted to medical, and 310 to surgical cases; of these 310 beds, about 190 are exclusively appropriated to cases of accident. In the year 1858, the hospital received 27,790 patients, including 3,976 in-patients and 23,814 out-patients. The accidents brought into the hospital, during 1858, were 11,529, including 2,090 in-patients and 9,439 out patients.

The Middlesex Hospital, from recent enlargements, contains upward of 300 beds, of which 185 are for surgical, and 120 for medical cases. The cancer establishment receives 33 patients. Wards are specially appropriated to cases of uterine disease and of syphilis; 2,109 in-patients were admitted during the past year. The number of out-patients during the same period amounted to 16,469.

Royal Westminster Ophthalmic Hospital.—This hospital set the example in London, in 1816, of receiving the poor on their own application, without letters of recommendation. During 1857, 6,315 persons were treated, of whom 160 were admitted into the hospital, and 6,155 were treated as out-patients; of these, nearly 2,000 were children of tender age. The principal operations were—57 for hard cataract; 49 for soft cataract; 14 for the formation of artificial pupil; 220 for strabismus; 227 for the removal of tarsal tumors; 5 for the removal of deformity of staphyloma; 3 for the removal of tumor in the orbit; 2 for osteal abscess; 1 for extirpation of the eyeball, on account of malignant disease. In addition, several hundred minor operations were performed.

Royal Orthopædic Hospital.—The daily attendance of out-patients exceeds 100, the average number annually being 1,600; and the number admitted from the commencement exceeds 21,000. Out of this large number, it is stated, not one death has occurred under treatment, neither has there been any instance of permanent suffering or injury.

Lock Hospital, London.—Patients treated, from January, 1747, to 31st December, 1857, 74,389. In-patients cured from 31st December, 1857, to 31st December, 1858, 333; out-patients ditto, 2,187; in-patients, 31st December, 1858, 52; out-patients ditto, 269; died, 2—2,843. Making a total of 77,232.——Asylum.—Admitted from July, 1787, to 31st December, 1858, 1,555; restored to their friends since the opening of the institution, 309; placed in respectable service, ditto, 391; died, ditto, 22.

Glasgow Royal Infirmary.—When the buildings at present in progress are completed, the accommodation will be much increased. Number of beds, 600. During the year 1858 the number of in-patients treated was 3,500. Out-patients, 10,422 were treated at the dispensary. Operations during the year, 185; amputations, 60; excision of tumors, 32; excision of bones and joints, 8; reduction of dislocations, 23; lithotomy, 13; various, 49.

The Lying-in Hospital, Rutland Square, Dublin.—This hospital, established in 1745, and chartered by George II., in 1756, is the largest establishment of the kind in the British dominions, and contains 130 beds, 15 of which are appropriated to the diseases of females. About 2,000 women are annually received into the institution.—*London Lancet.*

